# Land Use and Food Intake of Future Inhabitants: Outlining a Representative Individual of the Most Exposed Group for Dose Assessment

**DOI:** 10.1007/s13280-013-0400-z

**Published:** 2013-04-26

**Authors:** Peter Saetre, Jack Valentin, Per Lagerås, Rodolfo Avila, Ulrik Kautsky

**Affiliations:** 1 Swedish Nuclear Fuel and Waste Management Co. (SKB), Box 250, 101 24 Stockholm, Sweden; 2 Öregrundsgatan 15, 115 59 Stockholm, Sweden; 3 Swedish National Heritage Board (RAÄ), UV Syd, Odlarevägen 5, 226 60 Lund, Sweden; 4 Facilia AB, Gustavslundsvägen 151G, 167 51 Bromma, Sweden

**Keywords:** Safety assessment, Radiological protection, Ecosystems, Ingestion, Self-sustained communities

## Abstract

The radiation doses to humans resulting from a potential release of radionuclides from a geological repository for long-lived waste are assessed over tens or even hundreds of thousands of years. Ingestion is expected to be the major exposure pathway, and the group with the highest exposures will be those that consume the most contaminated food. In this paper, we characterize the group of individuals with the highest exposures by considering the physical and biological characteristics of the contaminated area and human requirements for energy and nutrients. We then calculate intake rates based on land-use scenarios drawn from self-sustained communities spanning prehistoric times to an industrial-age agrarian culture. The approach is illustrated by simulating groundwater release of four radionuclides to an expected discharge area. We argue that the derived intake rates may serve as credible bounding cases when projected doses are evaluated for compliance with regulatory criteria.

**Electronic supplementary material:**

The online version of this article (doi:10.1007/s13280-013-0400-z) contains supplementary material, which is available to authorized users.

## Introduction

The radiation doses to humans resulting from a potential release of radionuclides from an existing or planned repository for long-lived waste are assessed over tens or even hundreds of thousands of years. The assessment involves estimating activity concentrations of radionuclides in the environment (air, soil, sediment, and water) as well as in plants and animals that may serve as food for human inhabitants (Avila et al. 2013). Assumptions as to the characteristics and habits of the humans that will inhabit or utilize the contaminated areas are required in order to estimate external exposures and intakes of contaminated food (i.e., exposure scenarios have to be defined). The resulting dose, through all pathways of exposure and intake, can then be computed using dose coefficients (dose per unit exposure; dose per unit intake taking account of radionuclide retention in the human body and exposure from progeny radionuclides). In a safety assessment, the final step is to compare the computed doses with various dose criteria (ICRP 2006; Kautsky et al. 2013).

For the purpose of protection, the potential exposure is evaluated for a person representative of the more highly exposed individuals in the population (i.e., the most exposed group as explained in ICRP 2006). Due to the long time-scales considered for evaluating the safety of disposal of long-lived solid radioactive waste, the characteristics and habits of future inhabitants, as well as the characteristics of the contaminated area, can only be based on a number of assumptions (cf. ICRP 1998). Consequently, the representative person is a hypothetical construct. The assumed habits should reflect all relevant pathways of exposure and, as far as can be ascertained, be reasonable and sustainable with respect to the considered area, as well as human physiological requirements (ICRP 2006). Thus, by identifying environmental media accessible to humans and with a high potential for radionuclide accumulation, and then considering human behavior that would lead to the greatest exposure from utilizing such contaminated natural resources, a framework for calculating stylized exposure scenarios can be defined.

For radioactive material that reaches the surface ecosystems from a geological repository, the exposures from ingestion of contaminated food and water are expected to far exceed those resulting from inhalation or external exposure. For example, consumption of natural foods from an aquatic ecosystem contaminated with radioactive carbon (^14^C) is expected to be a dominant source for exposure from the existing and planned geological repositories for low and intermediate radioactive waste (Bergström et al. 2008). Similarly, consumption of crops from cultivated peat soils contaminated with radium (^226^Ra and radioactive progeny) is likely to be a major exposure route for a geological repository for spent nuclear fuel (SKB 2010, 2011; Avila et al. 2013).

In this paper, we describe a method for identifying the most exposed group with respect to the potential exposure from a geological repository for long-lived, high-level radioactive waste (specifically spent nuclear fuel) which is planned in Forsmark, Sweden. The paper focuses on the intake via food per unit contamination, i.e., how human characteristics and habits will affect the amounts of radionuclides that are ingested with food. However, the principles adopted can also be applied to identifying a representative person for other exposure routes (e.g., drinking water, inhalation, and external exposure). In order to assess intake from contaminated food, we consider different possible uses of land and natural resources, given the constraints set by the physical landscape and human requirements for energy and nutrients. Assumptions on habits are drawn from self-sustained low- and higher-technology societies from prehistoric times to an industrial-age agrarian culture. Thus, we identify stylized exposure scenarios, based on cultures and habits with high intake rates per unit radionuclide concentration. We recognize that the assigned characteristics and habits are unlikely to provide a realistic estimate of the actual characteristics and habits of human beings in the far future, but argue that the scenarios may serve as credible bounding cases when projected doses are evaluated for compliance with regulatory criteria.

## Initial Considerations and Central Concepts

Geo-hydrological modeling suggests that contaminated groundwater from a geological repository in a coastal area flows primarily to low points in the landscape, e.g., along the shoreline and in shallow parts of sea basins, or to lakes, streams, and mires when the discharge is to the terrestrial environment (Berglund et al. 2013). Radionuclides that enter surface ecosystems may accumulate in sediment and soil layers and will contaminate surface water and air. Contaminants that enter mires and aquatic food webs will accumulate in organisms that can be used as food by human inhabitants. When contaminated mires are used for haymaking, or are drained and cultivated, radionuclides will end up in crops and in livestock meat and dairy products. Ingestion of contaminated food or water will cause internal radiation exposure, and inhalation of contaminated air and external radiation from the environment may cause additional exposures.

Considering the ingestion route, the most exposed group will be the future inhabitants who consume the most contaminated food and water. If the most contaminated area of the landscape accessible to human use can be identified, and the concentration of radionuclides in food produced on this land can be calculated, then identifying a person representative of the most exposed group can be seen as a two-step process. The first step comprises identifying the type of community that would utilize contaminated natural resources in a way that would maximize exposure, and the second step comprises estimating reasonable fractions of the contaminated food items in the diet of the most exposed individuals in such a community.

For radionuclides with a high degree of bioaccumulation in aquatic or mire food webs, we expect the highest intake rate of contaminated food to occur among self-sustained hunter-gatherer communities. However, when radionuclides have accumulated in sediments, draining and cultivating the contaminated land may result in the highest intake rates of contaminated food (Table 1).

**Table 1:** Exposure through ingestion for ecosystems expected to receive direct or indirect releases of contaminated groundwater from a geological repository. Environmental media and examples of contaminated food items are listed together with self-sustaining human cultures associated with habits and land-uses expected to result in high intake rates

Ecosystem	Environmental media	Pathways	Food items	Most exposed group
Sea	Sea water and surface sediments	Marine food web	Fish	Fisherman, Hunter-gatherer
Lake/stream	Lake water and surface sediments	Fresh water food web	Fish, Crayfish	Hunter-gatherer
Mire	Mire sediments (peat)	Mire food webs	Fungi, Berries, Game	Hunter-gatherer
	Peat/agricultural soil	Haymaking/fertilization	Meat, milk/crops	Farmer
Arable	Agricultural soil	Draining and cultivation of peat	Meat, milk, crops	Farmer

The physical landscape governs and sets the limits for human use of land and natural resources. However, human activity may transform the natural landscape (including the most contaminated area) to a cultural landscape, e.g., by conversion of forests or mires into arable land or meadows. Consequently, the number of individuals that can be supported by natural resources from a unit area of land or water will depend on both land-use practice and available technology (Box S1 in Electronic Supplementary Material).

## Outlining the Most Highly Exposed Individuals

The extent to which a contaminated area of a natural or cultural landscape can support the food demand of future human inhabitants depends on the physical properties of the landscape, the number and characteristics of the exposed individuals, technology at hand, and cultural preferences. Below we sketch out detailed principles for calculating intake rates of radioactively contaminated food for a person who is representative of the most exposed group. For convenience with respect to dose calculations, food intake rates are expressed as a fraction of the yearly energy demand throughout (Box S2 in Electronic Supplementary Material). Assumptions on group size, land use, productivity, and diet are drawn from self-sustained societies from prehistoric times to an industrial-age agrarian culture (see Electronic Supplementary Material for a historical background), considering the likely size of the most contaminated area and human physiological constraints with respect to energy and nutrients.

### Prehistoric Hunter-Gatherers

Stone-age cultures from the Mesolithic and middle Neolithic periods serve well as a local reference for the most exposed group with respect to contamination from intake of marine and terrestrial natural food (see background discussion in Electronic Supplementary Material).The future landscape in Forsmark under conditions of weak human influence would most likely be a mosaic of forests, mires, and lakes, with a decreasing influence of the sea as the shoreline transgresses with time (Lindborg et al. 2013). One principle for calculating a diet of individuals inhabiting a future landscape is to assume that self-sustained communities make use of available resources in the home range in proportion to their production (Avila et al. 2010). However, there are many factors besides potential production that affect foraging behavior in a patchy landscape. For example, the distance to, and the harvesting/capturing time associated with, available food items will affect the foraging economy (i.e., the net energy intake). Storage efficiency will modify the yield, and cultural preferences and nutritional needs will ultimately determine the diet (Hassan 1975).

The most exposed individuals would be the ones making full use of the contaminated parts of the landscape. Thus, if contaminants were restricted to the sea, the most exposed group would be one with a marine diet, whereas if the contaminated groundwater primarily reached a lake with surrounding mire, the most exposed group would be found in a community with a mixed diet. From the historical record of hunter gatherers it is reasonable to assign a social structure that encompasses 40 members (and a corresponding forage area of 200 km^2^) also to future foragers (Marlow 2005 and discussion in Electronic Supplementary Material). With these assumptions most of the central investigation area of Forsmark (including all potential discharge areas for contaminated groundwater) would be within the home range of practically any settlement on the coast.

A simple way to estimate an upper limit for consumption of contaminated food items is to assume that the most exposed group consumes *all* food items that they can reasonably harvest from the contaminated area, given constraints on demands for energy and nutrients. As directly contaminated mires and lakes are expected to be limited in size, food items produced in these areas will only make up a fraction of the diet consumed by a typically sized hunter and gatherer group. Consequently, the residual dietary demands of inhabitants can only be fulfilled by intakes of uncontaminated food, and human needs for carbohydrates, fat, and protein do not need to be explicitly addressed when calculating intake rates of contaminated foods.

When contaminated groundwater reaches a sea basin close to the shore a considerable area can become contaminated. From archeological records, it can be inferred that marine proteins frequently made up all of dietary protein intake for hunting and gathering communities living on the coast. However, as the fish of the Baltic coast typically has a very low fat content [e.g., perch (*Perca fluviatilis*), pike (*Esox lucius*), and cod (*Gadus morhua callarias*)], there is an upper boundary as to how much contaminated fish could possibly be consumed. Excessive protein consumption may lead to toxic effects due to elevated levels of amino acids, ammonia, and insulin (causing nausea and diarrhea), and thus the upper level for healthy consumption of protein is 25 % of the total energy intake (Bilsborough and Mann 2006). Given a total energy demand of 3000 kcal/day, and an energy content of protein of 4 kcal/g, the maximum safe yearly consumption is 68 kg of proteins. With a protein to carbon ratio of 1.8 g/g C (perch, pike, and cod), this corresponds to a maximum yearly fish consumption of 38 kg C, or 35 % of the yearly carbon consumption. However, it should be noted that this upper boundary reflects physiological constraints on present-day man in combination with characteristics of the site, and it is not applicable to consumption of fish rich in fat (e.g., salmon species).

### The Infield-Outland Farming System

Mires and wet meadows have been an important resource for animal fodder from the last millennium BC, and the dependence on willow and sedges probably increased with the development of efficient tools. Thus a self-sufficient Iron-Age family serves as a historical reference for the most exposed group with respect to exposure from wetland hay through infield-outland farming. By combining the ecological basis of production and the physical features of the landscape (including distance), the land-use pattern of Iron-Age farming has been simulated for family-sized groups (Widgren 1979). These computer-generated land-use patterns correspond surprisingly well with observations of field boundaries and dwelling sites from the period in Östergötland (South-Eastern Sweden). Although there is no need to model explicitly the spatial structure of an Iron-Age settlement in the context of a safety assessment, the principles behind the calculations of land use and food consumption are highly relevant. Thus, by using the need for manure to fertilize an infield system and the production of fodder required to support the corresponding livestock through the winter period [15 000 kg dry weight (dw), Table 2]. Widgren (op. cit.) estimated that the ratio between arable land, meadow and pasture would be approximately 1:10:10. He then used literature data on productivity to calculate the production of cereal, meat, and dairy products, and their contribution to the Iron-Age diet (Table 2).

**Table 2:** Land use and consumption of an Iron-Age family farm, corresponding to 6 adult individuals. Calculations assume a mixed herd of livestock (7 cows, 3 heifers, 3 oxen, 5 pigs, and 16 sheep) with milk cows making up less than half of the animal stock. Updated from Widgren (1979)

Land use	Area (ha)	Food item	Productivity (kg year^−1^ m^−2^)	Calorie content (kcal/kg)	Yearly production (kcal)	Daily consumption (kcal/individual)
Arable	3	Cereal	0.05	3200	4 800 000	2192
Meadow	30	Dairy products^b^	0.0047	400	1 116 000	510
Pasture^a^	30	Meat^b^	0.0005	2000	600 000	274
						2975

^a^Minimum area of pasture assuming primary productivity as meadow (500 kg hay/ha)

^b^Meat and dairy product productivity expressed per unit area of pasture and meadows combined

In an Iron-Age infield-outland system, cattle would typically graze pastures and forest on solid ground, whereas hay would be collected from meadows on solid ground and from mires. Thus, a self-sufficient Iron-Age agricultural community of two households could be used as a model for the most exposed group with respect to exposure from contaminated hay and manure for infield cultivation. An upper boundary for exposure would be to assume that the most exposed group fully depended on the contaminated mire for winter fodder. Wet grasslands are more fertile than dry ones, and mires in Central Sweden may produce up to 2400 kg dw grass and sedges per hectare (ha) and year (Borgegård 1994). A mire of approximately 10 ha could thus provide winter fodder and much of the manure needed for a few farming households. Most mires developing on discharge areas for deep groundwater in Forsmark are expected be larger than 10 ha at some time, but if the contaminated area was smaller, then the contaminated hay would be diluted by production from uncontaminated land.

### Agriculture in the Industrial Period

**Fig. 1:**
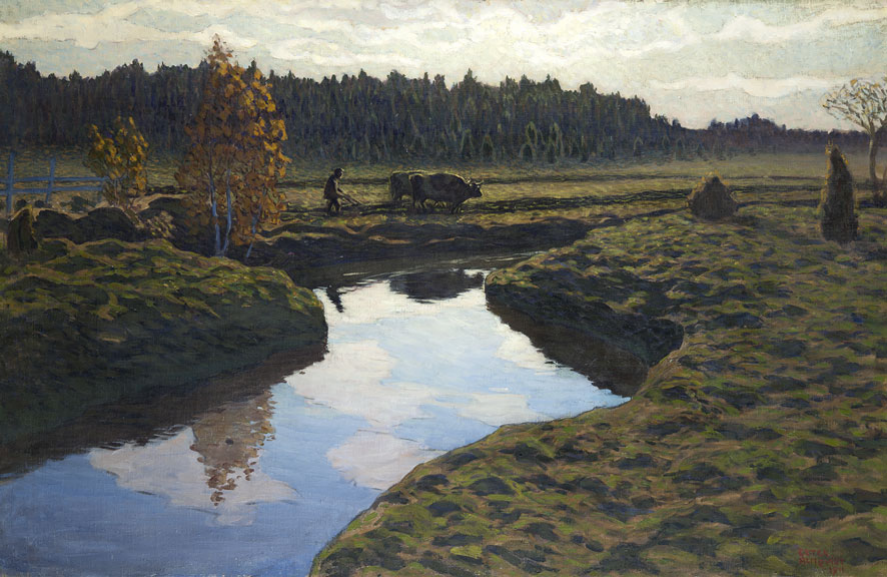
Cultivation of a drained mire. ‘Autumn Ploughing in the Marshland’, painting by Ester Almqvist 1911 (photo Hossein Sehatlou). Gothenburg Museum of Art

In the wake of the industrial revolution agricultural was modernized, and the demand of land led to large-scale draining and cultivations of lakes and mires. At the turn of the nineteenth century the majority of the farming population was found on self-sufficient small-scale farms, and we propose that these are a good reference for the most exposed group with respect exposure from draining and cultivating contaminated lake–mire systems (Fig. 1, and see Electronic Supplementary Material).

A common practice in Sweden around the turn of the nineteenth century was to cultivate the same crop for a few years, followed by a longer period without crop cultivation (i.e., rotations of green fodder or fallow). A large part of the arable land was used to produce fodder for animals. In addition to green fodder production, as much as two thirds of the cereal production may have been used as animal fodder (Morell 1998).

Animals were used as beasts of burden as well as for production of meat and dairy products. Statistics on livestock from the early twentieth century suggests an animal density of approximately 1 cow (or horse) per 1 ha arable land and farms without animals would have been very rare. Cattle were the most common type of livestock and the production of milk (on a wet weight basis) was an order of magnitude larger than the production of meat (cattle, pig, sheep, and horse combined; Morell 1998).

The practice of setting aside part of the arable land for small-scale cultivation of vegetables and peas started during this period or possibly a few centuries earlier. However, according to official statistics, only 1 % of all arable land was used for growing peas/beans, and Morell (1998) states that a fair fraction of this land would have represented contract cultivation in Southern Sweden (i.e., not for local consumption). Moreover, peas and beans do not grow well on soils rich in organic matter.

**Fig. 2:**
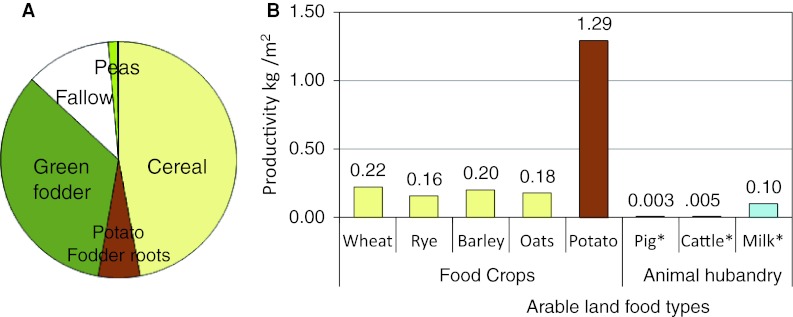
Use of agricultural land (**a**) and corresponding productivity (**b**) at the turn of the nineteenth century in Sweden. The *numbers* are based on official national statistics from the Swedish Board of Agriculture. The productivity from animal husbandry (*) has been calculated with respect to the area of green fodder and two thirds of the area used for cereal and root crop production, respectively

Farming practice must have varied regionally and locally, but for the purpose of a safety assessment national agricultural statistics from the turn of the nineteenth century can be used to sketch out typical land use and productivity for industrial-age agriculture (Fig. 2). Assuming that agriculture supplied the bulk of the diet for a farm household (i.e., that berries, game, and fish were just a limited dietary supplement), food intake would then be roughly proportional to the production of each food item on the farm.

As small family farms were dependent on the production of a limited area of land, we suggest that they serve well as a model for the most exposed group. Although peat soils were traditionally used for fodder production, cereals (oats, barley, and in particular rye) and potatoes also grow well on organic soils, and thus it can cautiously be assume that the most exposed group used organic soils for production of all bulk food.

Using an annual demand of 110 kg C per individual, a theoretical minimum support area of 6 ha can be calculated for 10 adult individuals, which means that a few farming households could most likely be fully supported by a contaminated mire which was drained for cultivation. Nevertheless, if the contaminated mire were smaller than this support area, the fraction of food items that would be contaminated could easily be adjusted to account for this.

## Results and Discussion

**Fig. 3:**
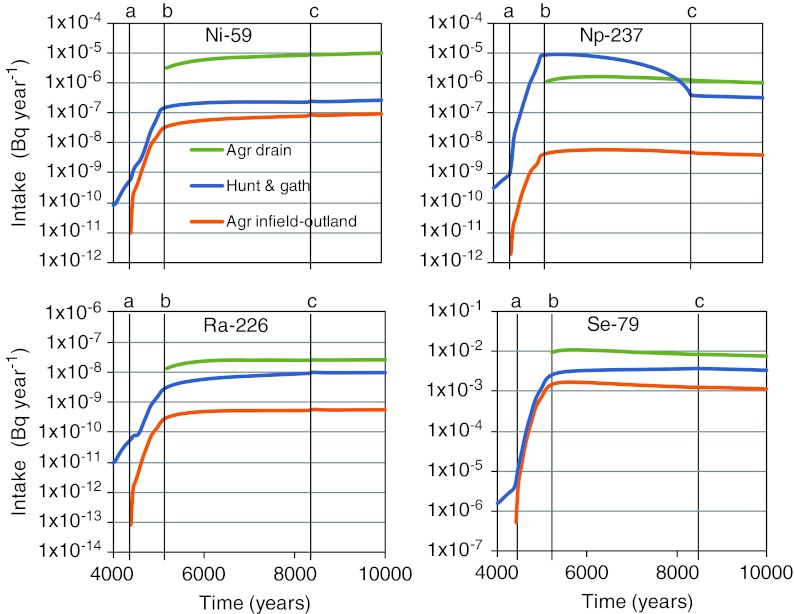
Intake rates (Bq year^−1^) of contaminated food given a unit release rate (1 Bq year^−1^) of contaminant for three land-use scenarios (*green* agriculture in the industrial period, *blue* hunter-gatherers, *red* infield-outland farming system) and four radionuclides (*top left* Ni-59, *top right* Np-237, *bottom left* Ra-226, *bottom right* Se-79). The results are based on simulations of one discharge area that develops from a sea basin into a lake–mire ecosystem. *Vertical lines* represent the start of lake isolation (*a*), time when discharge area is 2 m above sea level (*b*), and the time when the mire has grown to fully cover the original lake basin (*c*)

The exposure from consumption of contaminated food for a representative individual of the most exposed group is illustrated with a simulation. Four radionuclides are released at a constant rate (1 Bq year^−1^) to a potential discharge area in the future Forsmark landscape (Fig. 6 in Lindborg et al. 2013). During the simulation period which represents an interglacial interval (here, 9000 bc to ad 10 000), the discharge area develops from a sea basin, through a lake–mire complex, to a mire where the lake basin has been filled with sediments and peat. The activity concentrations in sea and lake water and in peat and agricultural soils were dynamically simulated (Avila et al. 2013). Equilibrium concentrations in natural food, fen hay, and agricultural products were then combined with the three outlined land-use scenarios, to calculate the yearly intake rates of radionuclides, averaged over 50 years (Fig. 3).

The results clearly show that radionuclide properties, the development of the surface environment, and the land-use scenario, all have significant effects on the intake rate of activity. For all four radionuclides, the highest intake rate was recorded after lake isolation, and for three of the radionuclides (^59^Ni, ^226^Ra, ^79^Se), cultivation of contaminated peat yielded the maximum intake rate. However, for ^237^Np which accumulates strongly in aquatic food webs, foraging the lake for natural food resulted in the peak intake rate of the radionuclide. In the following sections these patterns are examined and discussed in more detail.

### Coastal Hunting and Gathering Communities

The Forsmark area is expected to be covered by sea during a large fraction of an interglacial (Näslund et al. 2013), and during this period consumption of contaminated fish is the primary exposure pathway for ingestion. As the maximum sustainable yield of fish from the sea basin exceeds the safe protein consumption for 40 individuals (1300 kg C/year), the fraction of contaminated food (*f*_cont_) is determined by human physiological constraints with respect to protein toxicity (i.e., the carbon fraction is 0.35 for fish). However, the size of the contaminated sea basin is ultimately reduced by land rise (Lindborg et al. 2013) and in this example the fraction of contaminated fish in the diet will consequently decrease from approximately ad 4000.

The isolation of the lake from the sea spans 650 years, starting around ad 4400 (a in Fig. 3). At this time, the discharge area develops into a mire complex, where the mire eventually covers the entire lake basin (c in Fig. 3). To calculate the maximum exposure for hunters and gatherers with a mixed diet, all the sustainable production of natural food items in the contaminated area is assumed to be harvested. As the lake–mire system is much smaller than the sea basin, the yearly production of contaminated food only covers a fraction (~10 %) of the energy demand of a typical-sized hunting and gathering community (Table S1a in Electronic Supplementary Material). Consequently the bulk of food will be captured or harvested from uncontaminated parts of the landscape even for the most exposed group.

The production capacity of the lake–mire system is fairly constant over time and the increasing intake of radioactivity during lake isolation (a and b in Fig. 3) reflects rising activity concentrations in water and peat due to a reduction in water exchange. After lake isolation (~ad 5000) activity concentrations in the contaminated area approach long-term steady-state conditions, but the production of aquatic food decreases as the lake area declines. Thus, for a radionuclide where the primary source of exposure is aquatic food, intake rates decrease in response to the expansion of mire vegetation (see ^237^Np in Fig. 3).

The fraction of contaminated food of the most exposed group is a cautious upper bound for estimating the intake of radionuclides. The contaminated area is selectively harvested and, from a landscape perspective, the contaminated food is clearly over-represented in the diet. For example, during most of the sea stage all fish consumed is assumed to have been captured in the contaminated basin, although this basin contributes less than 20 % of the total marine resources available in a 200 km^2^ home range. Similarly, 10 % of the dietary carbon originates from the contaminated lake–mire complex, but the production from the contaminated area only makes up 1 % of available land resources in the home range.

### Agricultural Communities

Once the discharge area has emerged out of the sea it develops into a lake–mire complex, where contaminated mire grasses and sedges provide an exposure pathway for human settlers (~ad 4500, a in Fig. 3). Sustainable cultivation of peat is not possible in the young lake–mire system. However, when the discharge area has emerged 2 m above sea level (~ad 5200, b in Fig. 3) salt-water intrusions due to regular flooding at high sea levels are unlikely to occur, and draining and cultivation of contaminated peat is considered a relevant exposure pathway.

Following lake isolation, activity concentrations of the studied radionuclides are fairly stable in mire peat and vegetation, and the concentrations in agricultural soils, fodder, and food items vary little within the agricultural systems. Given industrial-age farming, two family farms can be supported by the contaminated mire from the point in time when sustainable cultivation is possible, whereas the demand for winter fodder of the infield-outland system can be supported at approximately ad 6000 (Table S1b in Electronic Supplementary Material). Thus, from these points in time there is no dilution in intake rates relating to the size of the contaminated area.

Comparing the two agrarian land-use scenarios, it is clear that intake of radionuclides is higher in the industrial-age system than in the infield-outland system (Fig. 3, all radionuclides). This primarily reflects that draining and oxidation of peat yields higher activity concentrations in agricultural soil than in the original mire or in soil contaminated through a steady input of organic fertilizer. However, the difference clearly varies (from a factor 3 to 100) with radionuclide properties such as half-life, tendency for soil sorption, and degree of bioaccumulation.

In both agricultural scenarios, the bulk of the energy demand of future inhabitants is assumed to be covered by agricultural production. The overall nutritional contents of diets from an infield-outland and an industrial-age agricultural system were very similar with respect to proteins, fat, and carbohydrates: 17:25:58 (% energy) and 16:24:60 (% energy), respectively. Though these diets are no more than the results from highly stylized calculation scenarios, the diets are fairly balanced and meet the lower value of acceptable macronutrient requirements for both fat (15 % energy, FAO 2010) and proteins (0.86 g/kg day, WHO 2007), and thus appear reasonable from human nutritional demands.

### Contrast with Present-Day Consumption Statistics

So far we have sketched out three stylized exposure scenarios, which may serve as bounding cases for intake rates of contaminated food. We argue that each one of these scenarios constitutes a reasonable bounding case with respect to the identified exposure pathways. The degree of caution that we have introduced can be further illustrated by contrasting the fraction of contaminated food in the proposed land-use scenarios with upper limits derived from a modern average diet, reflecting present-day food consumption patterns.

For this comparison, we have used a recent summary of Swedish consumption statistics, published by the Swedish Board of Agriculture (Wikberger and Johansson 2006). The relative contributions of major food types were first scaled in terms of yearly carbon consumption (Table S2 in Electronic Supplementary Material). We then used the fraction of unprocessed food (fresh or frozen) as a reasonable upper limit for the potential contribution of contaminated food (i.e., the production of food items on the scale of a discharge area was assumed to make up an insignificant fraction of raw material for the food industry).

From the comparison it becomes clear that the consumption of both natural and agricultural contaminated food items are at least five times higher in the historic land-use scenarios, as compared with upper limits based on recent food statistics. That is, for the historical and present-day calculation cases the fraction of contaminated food is 0.10 versus 0.02 for natural food items, and 1.0 versus 0.16 for agricultural food items (Table S1a, b vs. Table S2 in Electronic Supplementary Material). Moreover, this relationship does not change significantly if we assume that the total fraction, rather than the non-processed fraction, of the food items is a more reasonable upper limit for consumption of contaminated food today.

SKB is presently addressing the technical limitations in the SR-Site biosphere model as part of the ongoing development of its biosphere assessment methodology. Thus we aim to include more details on agricultural crop system (e.g., in terms of plant uptake and water balance) in future assessment models, so that we can estimate crop system-specific activity concentrations in agricultural produce from similar initial conditions.

## Conclusion

In this paper we have sketched out three land-use scenarios that may serve as bounding cases for intake of contaminated food, using three self-sustained historical societies as reference points. From our analysis we conclude that these constructs define a reasonable set of exposure scenarios for food ingestion, when projected doses are to be evaluated for compliance with regulatory criteria in an appropriate context (groundwater contamination for a coastal Scandinavian site). That is, the scenarios are likely to cover the most exposed group regardless of type of community existing at the site in the future.

We believe that consistency and compatibility between estimates of projected doses would benefit from a more explicit framework for identifying exposure scenarios, and hope that the principles outlined in this paper can contribute to this development. However, we recognize that the assigned characteristics and habits of future humans are unlikely to provide a very realistic estimate of the actual characteristics and habits of human beings in the far future. For this and several other reasons, projected doses in the far future should not be used to predict future health effects (ICRP 2006).

Ingestion is the expected major pathway of exposure from a geological repository, and the most highly exposed individuals will be those that consume contaminated food (and water). The relative contributions of different land-use scenarios to exposure are likely to vary with the properties of released radionuclides, with time and with the stage of development of a contaminated discharge area. Thus, it seems rational to evaluate several plausible sets of land-use scenarios in parallel, when assessing the radiological safety of future generations. We recommend that each such scenario should conform to the physical and biological constraints of the contaminated area, as well as the physiological and nutritional needs of human inhabitants. Moreover, land use should be sustainable and reasonable in the sense that there are historical records supporting the construct, or that it can be observed in existing human cultures.

## Electronic supplementary material

Below is the link to the electronic supplementary material.

Supplementary material 1 (PDF 213 kb)
