# Supplementary material for: Land Use and Food Intake of Future Inhabitants: Outlining a Representative Individual of the Most Exposed Group for Dose Assessment
Source: Ambio. 2013 Apr 26;42(4):488–96. doi: 10.1007/s13280-013-0400-z (PMC3636367; doi:10.1007/s13280-013-0400-z)
Supplement: Supplementary file 1 — Supplementary material 1 (PDF 213 kb) [file 13280_2013_400_MOESM1_ESM.pdf]

Electronic Supplementary Material

Land Use and Food Intake of Future Inhabitants – Outlining a Representative Individual of the Most Exposed Group for Dose Assessment

Peter Saetre, Jack Valentin, Per Lagerås, Rodolfo Avila, Ulrik Kautsky

HISTORICAL LAND USE IN CENTRAL SWEDEN

In this section, we give a brief historical background to land use associated with parts of the landscape where humans may be exposed to accumulating radionuclides. The examples date from prehistoric times, through early agriculture, to agriculture in the industrialized era.

Prehistoric hunter-gatherers

Remains from Mesolithic and Neolithic communities in Central Eastern Sweden have primarily been found from excavations at locations near the coast line at the time of the settlement (Knutsson and Knutsson 2003). Stable isotope analysis of animal and human bones suggests that marine food was the dominant protein source for many Mesolithic settlements, and that this was also true for some coastal settlements well into the Neolithic period (Lidén et al. 2004; Fornander et al. 2008).

Animal bone remains and stable isotope analysis show that food practice on Öland varied extensively in prehistoric times (Fig. S1; Eriksson et al. 2008). During the middle Neolithic period, settlers with a marine diet co-existed with communities where the marine protein diet was clearly supplemented with terrestrial protein sources (see KOP MN, RES MN and TOR MN in Fig. S1). The remains from these and other sites along the Swedish coast (e.g., Larsson 1988; Karsten and Knarrström 2003) are thought to reflect hunting-gathering communities, with foraging behavior varying from a preference for marine prey, over a mixed diet, to a diet where protein is dominated by terrestrial game.

Following the discussion above, stone-age cultures from the Mesolithic and middle Neolithic periods could serve as a reference for the most exposed group with respect to contamination from marine and terrestrial natural food items. Characteristics of the size of hunter-gatherer groups, their typical home ranges, and their mobility are hard to reconstruct from archaeological records and few such communities exist today. However, these characteristics can be studied from historic ethnographic records and related to habitat characteristics (e.g., biomass of primary producers) and diet (Marlow 2005).

From such analyses of more than 300 hunter-gatherer societies, it can be seen that the population density increases with plant biomass, leveling off at approximately 0.2 individuals per km^2 above a biomass of 30 kg/m^2. The typical size of a community at these biomass levels is just below 40 individuals, corresponding to a home range of approximately 200 km^2. The home range is the area an individual occupies over a whole year. However, for historical hunter and gatherer communities the camp site was typically moved within the home range several times a year (Marlow 2005).

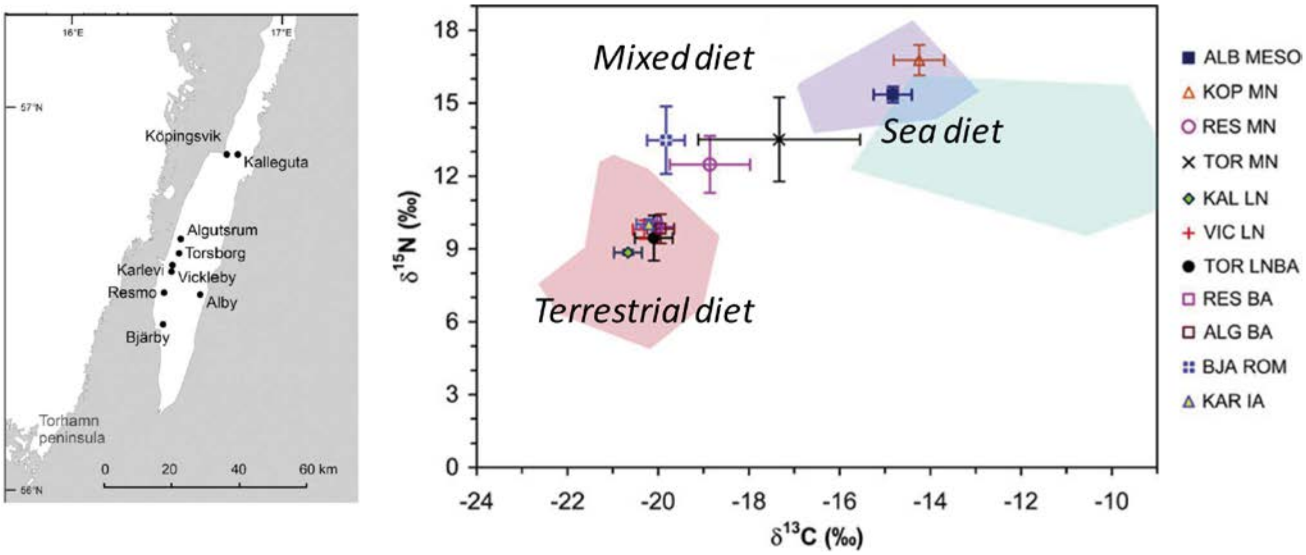

Fig. S1 Mean isotopic values and standard deviations for human bones representing different sites and prehistoric cultures on Öland. Colored fields represents predicted isotopic ranges for humans subsisting completely on herbivores (pink), marine mammals (dark blue), and marine fish (light blue). The first three letters in the symbol legends represents the site on the map (left). MESO, Mesolithic; EN, MN, LN, Early, Middle and Late Neolithic; BA, Bronze Age; ROM, Roman Period; IA, Iron Age. Modified from Eriksson et al. (2008)

The infield-outland farming system

During the last millennium BC the farming system and cultural landscape in Scandinavia went through a number of changes. Before this period, farming systems had made extensive use of natural resources (e.g., pollarding of trees and slash and burn agriculture). However, from the fifth century BC and onwards, the pollen records reflect large-scale changes in the landscape. These changes coincided with the appearance of houses with a section for livestock and the use of iron in cultivation and hay making tools.

During the Iron Age (500 BC – AD 1000), farms became stationary units with much higher productivity than previous agricultural systems. It is believed that the basis for the agrarian expansion was the integration of cultivation and cattle breeding. This agrarian system was characterized by fertilized arable fields and enclosed meadows (infield) on one hand and livestock grazing nearby pastures and forests (outlands) on the other hand. Hay making was also necessary to provide domestic animals with winter fodder.

The key principle behind this agricultural system was to fertilize the arable land with the nutrients from meadows and pastures, by the use of animal manure as organic fertilizer. This basic principle more or less characterized agricultural practices in Scandinavia well into the 18th century (Welinder et al. 1998)

In South and Central Scandinavia there are multiple pollen records that bear witness to the systematic transformations of mires, dominated by alder, into wet meadows dominated by willow and sedges from the last millennium BC, reflecting the increasing dependence on productive mire for cattle breeding (Berglund et al. 1991). The importance of mires to satisfy the demand for winter fodder probably increased with the development of efficient tools (e.g., the long-bladed scythe, c. AD 1000), and the practice was common well into the industrial period in Sweden. The social structure of farming units varied locally and regionally. However, the family appears to have been the basic unit for agriculture during much of the Iron Age, and there are archaeological records from Scandinavia of farms from the size of a core family to that of an extended three generations family (Lindquist 1974).

Agriculture in the industrial period

In the wake of the industrial revolution agricultural practice changed dramatically during the 19th century in Sweden. Modern crop rotation replaced previous practices, the use of new species and improved crops types and animal breeds were established, and improved methods for fertilization were introduced. A growing population increased the demand on food production. The amount of arable land increased and the importance of meadows declined dramatically, as fodder was now primarily produced on arable land. Although this modernization increased the trade in agricultural products and the dependence on cash crops, most farmers were still self-sufficient with respect to their daily food intake (Morell 1998).

It is during this period that we first observe the use of large-scale drainage to increase the land available for cultivation. The levels of large lakes were lowered (e.g., Hjälmaren and Tämnaren) and smaller lakes and mires were completely drained (e.g., Kvismaren and several lakes and mires along the catchments of Fyrisån and Olandsån). Whereas some of the more conspicuous changes in the landscape were made by large economic enterprises, large areas of mires in North Sweden were drained by local farmers (Morell 1998).

Sustainable use of organic peat that originates from drained mires is expected to be productive for agriculture during a limited period only. This is because peat subsides when groundwater is lowered, and an organic layer may be reduced by as much as 60% in thickness by compaction and oxidation within 100 years (Berglund 2008). However, from the perspective of the safety assessment, a period of 50–100 years is relevant for assessing the lifetime risk to one generation of the most exposed group.

At the turn of the 19th century the majority of the farming population was found on small-scale farms. Family farming which fully occupied all family members, and was sufficient to sustain an average household of five adults, typically required a minimum of 5 to 10 ha of arable land (Morell 1998).

PERTINENT FOOD STATISTIC

The contribution of contaminated natural food items to the total carbon intake of a representative individual of the most exposed group is presented in Table S1.

Table S2 provides typical daily intake rates and relative carbon contribution of different food items from recent Swedish food statistics.

Table S1 The contribution of contaminated natural food items (f_{cont}) to the total carbon intake of a representative individual of the most exposed group calculated from simulations of a potential discharge area for deep groundwater in Forsmark.

a) Hunter and gatherers foraging a contaminated coastal sea basin at AD 4000 (sea) or a contaminated lake/mire system at AD 5500 (mixed)

Ecosystem	Food item	Area (m^2)	Productivity (C; kg m^{-2} y^{-1})	f_{cont} Sea (-)	f_{cont} Mixed (-)
**Sea**	Fish	$6.9 \cdot 10^{6}$	$2.2 \cdot 10^{-4}$	0.34	.
**Mire**	Berries	$9.2 \cdot 10^{5}$	$1.3 \cdot 10^{-4}$	.	0.03
	Fungi	.	$1.2 \cdot 10^{-4}$	.	0.03
	Game	.	$8.3 \cdot 10^{-6}$	.	0.002
**Lake**	Fish	$6.4 \cdot 10^{5}$	$2.7 \cdot 10^{-4}$	.	0.04
	Crayfish	.	$3.1 \cdot 10^{-5}$	.	0.005
				**0.34**	**0.10**

b) Self sustained agrarian communities collecting hay from a contaminated mire (inland-outfield, AD 6000), or draining and cultivating contaminated peat (AD 5200)

Land use	Food item	Infield-outland			Cultivation of drained peat		
		Area (m^2)	Productivity (C; kg m^{-2} y^{-1})	f_{cont} (-)	Area (m^2)	Productivity (C; kg m^{-2} y^{-1})	f_{cont} (-)
**Green fodder**	Dairy products[a]	$10 \cdot 10^{4}$ [b]	$4.4 \cdot 10^{-4}$ [d]	0.24	$2.0 \cdot 10^{4}$	$6.5 \cdot 10^{-3}$ [e]	0.25
	Meat[a]	$50 \cdot 10^{4}$ [c]	$9.1 \cdot 10^{-5}$ [d]	0.05		$1.0 \cdot 10^{-3}$ [e]	0.04
**Crop**	Cereal	$5 \cdot 10^{4}$	$1.6 \cdot 10^{-2}$	0.71	$3.0 \cdot 10^{4}$	$6.7 \cdot 10^{-2}$	0.6
	Potato	.	.	.	$3.7 \cdot 10^{3}$	$1.0 \cdot 10^{-1}$	0.11
	Fallow	.	.	.	$7.7 \cdot 10^{3}$	.	.
				**1**			**1**

a) Dairy products and meat produced from common land/fodder
b) Wetland hay
c) Pasture
d) Productivity in terms of combined wetland and pasture area
e) Productivity in terms of combined area for green and crop fodder

Table S2 Typical intake rates of food from recent Swedish food statistics (Wikberger et al. 2006). For each food item the daily intake (kcal) is listed together with the relative carbon contribution (f_{diet}). The carbon contribution of non-processed food (fresh or frozen) has been used as the upper limit for intake of contaminated food (f_{cont}). Asterisks (*) indicate the contribution from non-agrarian food items that can be harvested in natural ecosystems in Central Sweden (e.g., game, fish, and berries)

Food item	Intake (kcal)	f_{diet} (C; kg/kg)	f_{cont} (C; kg/kg)
**Bread and cereals**	910	0.32	
**Meat and meat products**[a]	384	0.12	0.004*, 0.08
**Fish and crustaceans**[b]	66	0.02	0.01*, 0.002*
**Milk**	202	0.07	
**Cream**	68	0.02	
**Cheese**	150	0.05	
**Eggs**	36	0.01	0.01
**Margarine and oil**	232	0.06	
**Vegetables**[c]	76	0.03	0.02
**Fruit and berries**[d]	195	0.07	0.001*
**Potato**	147	0.05	0.05
**Sugar**	92	0.03	
**Other processed food**[e]	433	0.14	
		**1**	**0.017*, 0.16**

a) 64% non-processed, including 3% game.
b) 49% non-processed fish, 8% non-processed crustacean
c) 80% non-processed vegetables
d) 2% domestic berries (non-processed)
e) Coffee, cocoa, chocolate, candy, beer, soft drinks, etc.

BOX S1: CONCEPTUAL MODEL OF A NATURAL/CULTURAL COASTAL LANDSCAPE

The conceptual model in Figure S2 implies land use of three different self-sustained communities with increasing technological sophistication: a hunting-gathering community (Mesolithic /Neolitic), an infield-outland agricultural community (e.g., Iron Age), and an agrarian community with technology for draining and cultivating lakes and mires (early industrial age).

Under the agrarian scenarios, natural elements have been converted to cultural elements when possible, as limited by the physical landscape (e.g., soil type and height above sea level) and available technology (e.g., tools for draining and cultivation).

Landscape elements under a weak influence of human activity are represented by dark blue (coastal water available for fishing), dark green (forest), orange (mire), and light blue (lake/streams), whereas elements under strong human influence are represented by light green (meadows or mires used for haymaking) and yellow (arable land). Black dashed areas indicate the size of a typical support area (Welinder 1998) while red dashed lines represent the size of the most contaminated area in the landscape. The areas available for fishing and the size of catches also depend on the tools and techniques at hand (left side).

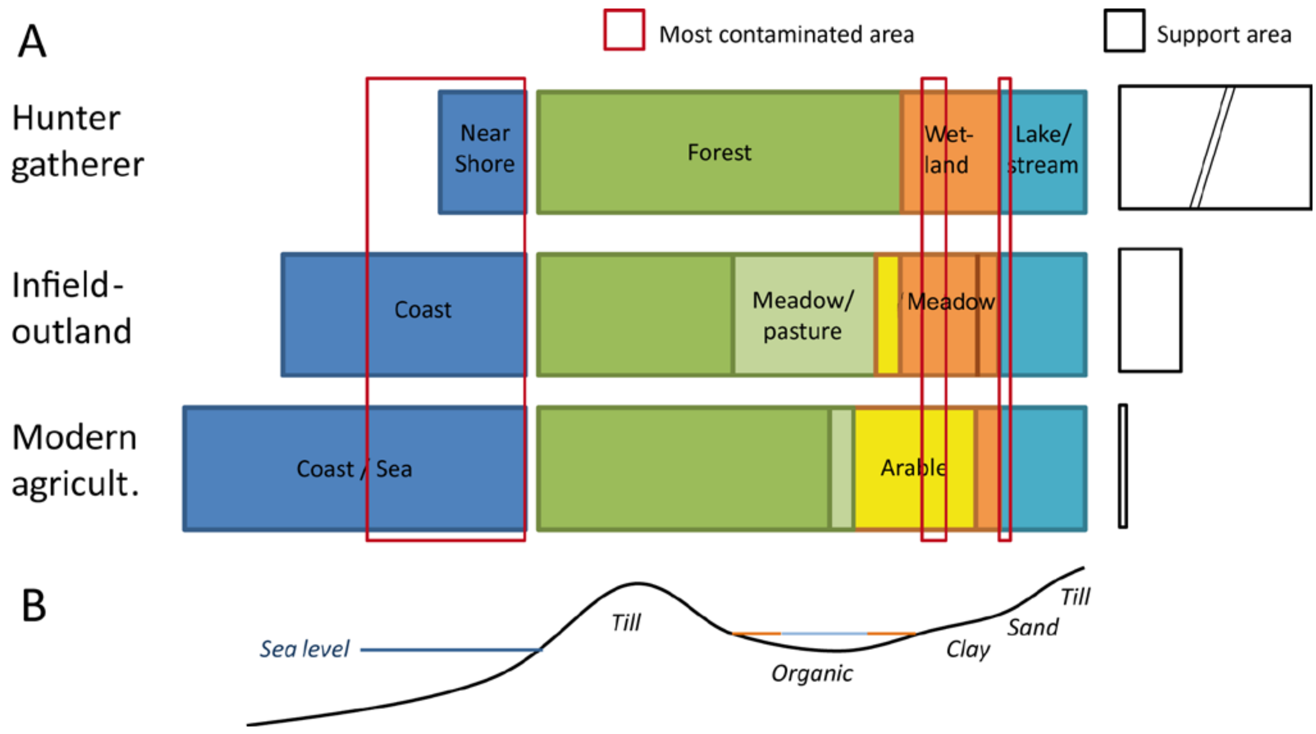

Fig. S2 Conceptual model of a natural/cultural coastal landscape. **A)** The stacked horizontal bars represent the total area of natural and cultural components in the landscape. **B)** Topography and surface soil types for a transect through a coastal landscape. Note that the transect and the cumulative area bars of the landscape elements above are not aligned

BOX S2: INTAKE RATE AND DOSE FROM INGESTION OF CONTAMINATED FOOD

The dose from consumption of food contaminated by radionuclides is determined by the food intake rate, the activity concentration of each food item consumed, and the dose coefficients for ingestion (Bergström et al. 2008). The annual dose (Sv) from exposure to radionuclide *i* can thus be calculated as:

$$Dose_i = Ing_C \cdot DosCoef_{ing_i} \cdot \sum_j f_{cont_j} \cdot Conc_j$$

where Ing_C (110 kg C) is the total food consumption (expressed as the annual carbon requirement; Avila and Bergström 2006), $DosCoef_{ing}$ (Sv/Bq) is the radionuclide specific factor for converting consumed activity to committed effective dose (ICRP 1995), f_{cont_j} is the fraction of the total carbon requirement satisfied by consumption of contaminated food item j, and $Conc_j$ is the activity concentration of food item j (Bq/kg C).

Moreover, the intake rate of radionuclide i is calculated by summing the product of the two last terms over all food items j.

For a set of contaminated food items, a representative person of the most exposed group can be described by the array $\overline{f_{cont}}$ maximising dose from food ingestion, given the constraints posed by the physical configuration of the landscape, a plausible land-use scenario and human nutritional needs.

REFERENCES

Avila, R., and U. Bergström. 2006. Methodology for calculation of doses to man and implementation in Pandora. Svensk Kärnbränslehantering AB, SKB R-06-68, Stockholm, Sweden, Report, 29 pp.

Berglund, B.E., L. Larsson, N. Lewan, E. G. A. Olsson, and S. Skansjö. 1991. Ecological and social factors behind the landscape changes. In *The cultural landscape during 6000 years in southern Sweden - the Ystad Project; Ecological Bulletins 41* (pp. 425-445), ed. B. E. Berglund, 495 pp. Hoboken, NJ: Wiley-Blackwell.

Berglund, K., 2008. Torvmarker en resurs i jordbruket igår, idag och även imorgon? In *Svensk mosskultur: odling, torvanvändning och landskapets förändring 1750–2000* (pp. 483-498), ed. L Runefelt, 528 pp.

Stockholm, Sweden: Kungliga Skogs- och Lantbruksakademien (in Swedish).

Bergström, U., R. Avila, P.-A. Ekström, and I. de la Cruz. 2008. Dose assessments for SFR 1. Svensk Kärnbränslehantering AB, SKB R-08-15, Stockholm, Sweden, Report, 176 pp.

Eriksson, G., A. Linderholm, E. Fornander, M. Kanstrup, P. Schoultz, H. Olofsson, and K. Lidén. 2008. Same island, different diet: Cultural evolution of food practice on Öland, Sweden, from the Mesolithic to the Roman Period. *Journal of Anthropological Archaeology* 27: 520–543.

Fornander, E., G. Eriksson, and K. Lidén. 2008. Wild at Heart: approaching Pitted Ware identity, economy and cosmology through stable isotopes in skeletal material from the Neolithic site Korsnäs in Eastern Central Sweden. *Journal of Anthropological Archaeology* 27: 281–297.

ICRP. 1995. Age-dependent doses to the members of the public from intake of radionuclides - part 5; compilation of ingestion and inhalation coefficients. ICRP Publication 72. *Annals of the ICRP* 26 (1).

Karsten, P., and B. Knarrström. 2003. *The Tågerup excavations*. Stockholm, Sweden: Riksantikvarieämbetet, 254 pp.

Knutsson, K., and H. Knutsson. (2003). Stone Age Transitions: Neolithization in central Scandinavia. *Documenta Praehistorica* XXX: 49-78.

Larsson, L. 1988. *Ett fångstsamhälle för 7000 år sedan: boplatser och gravar vid Skateholm*. Lund, Sweden: Signum, 191 pp (in Swedish).

Lidén, K., G. Eriksson, B. Nordqvist, A. Götherström, and E. Bendixen. 2004. The wet and the wild followed by the dry and the tame—or did they occur at the same time? Diet in Mesolithic—Neolithic southern Sweden. *Antiquity* 78: 23–33.

Lindquist, S.-O. 1974. The development of the agrarian landscape on Gotland during early Iron Age. *Norwegian Archaeological Review* 7: 6-32.

Marlow, F.W. 2005. Hunter-gatherers and human evolution. *Evolutionary Antropology* 14: 54-67.

Morell, M. 1998. *Det svenska jordbrukets historia – Jordbruket i industrisamhället 1870 – 1945*. Stockholm, Sweden: Natur och Kultur, 392 pp (in Swedish).

Welinder, S., E. A. Pedersen, and M. Widgren. 1998. *Det svenska jordbrukets historia – Jordbrukets första femtusen år 4000 f.Kr – 1000 e.Kr.* Stockholm, Sweden: Natur och Kultur (in Swedish).
